# Case report: An atypical case of uterine adenosarcoma with extensive bizarre stromal cells and amplification of MDM2

**DOI:** 10.3389/fonc.2024.1351646

**Published:** 2024-03-14

**Authors:** Xiaoling Xiao, Ying He

**Affiliations:** ^1^ Department of Pathology, West China Second University Hospital, Sichuan University, Sichuan, China; ^2^ Key Laboratory of Birth Defects and Related Diseases of Women and Children, Sichuan University, Ministry of Education, Sichuan, China; ^3^ National Health Commission (NHC) Key Laboratory of Chronobiology, Sichuan University, Sichuan, China

**Keywords:** polypoid lesions, adenosarcoma, bizarre stromal cells, MDM2, uterus

## Abstract

Polypoid lesions in the uterus are very common. There are a lot of benign lesions and malignant tumor should be considered. Adenosarcoma, one of the differential diagnoses, is a rare mixed epithelial and mesenchymal tumor consisting of benign epithelial components and sarcoma stroma. Here, we present a case of atypical adenosarcoma that has never been reported. This tumor was composed of benign endometrial epithelium and hyperplastic stroma with extensive bizarre stromal cells. But the cleft-like spaces and papillary stomal fronds which were the typical histological images of adenosarcoma were absent. These stomal cells, including bizarre cells, were positive for vimentin, CD10, ER, PR, cyclin D1 and P16 but were immunonegative for caldesmon. Furthermore, this tumor harbored amplification of MDM2, as revealed by fluorescence *in situ* hybridization testing (FISH) and next-generation DNA sequencing (NGS).

## Introduction

Polypoid lesions in the uterus are very common. Such as benign endometrial and endocervical polyps and adenofibroma, as well as various malignant tumors, including endometrial stromal sarcoma, adenosarcoma, carcinosarcoma, botryoid rhabdomyosarcoma, and other uterine sarcomas should be distinguished. In addition to malignant tumors, bizarre stromal cells are occasionally present in benign polyps, but these appear to represent a degenerative phenomenon, as the cells show smudged nuclear chromatin and are not mitotically active (1). Here, we present a case of atypical adenosarcoma with bizarre stromal cells accompanied by amplification of MDM2.

Müllerian adenosarcomas, were initially reported in 1974, are uncommon biphasic epithelial-mesenchymal tumors with benign epithelial elements and a malignant mesenchymal component (2). The lesion most often occurs in the endometrium, but rare tumors in the cervix, ovary, fallopian tube, and vagina have been reported (3, 4). The median age is 50-59 years old. Adenosarcoma is usually treated by hysterectomy and bilateral salpingo-oophorectomy; more conservative resection is occasionally performed for young patients. Most patients have good clinical outcomes. Moreover, aggressive clinical behavior often includes deep myometrial invasion and a pure sarcoma component comprising ≥25% of the tumor (5). The pathogenesis of uterine adenosarcomas is poorly understood. Molecular studies to date have revealed mutations in genes such as MDM2/CDK4, TP53, ATRX, FGFR2, KMT2C and DICER1 (6–9).

## Case report

**Figure 1:**
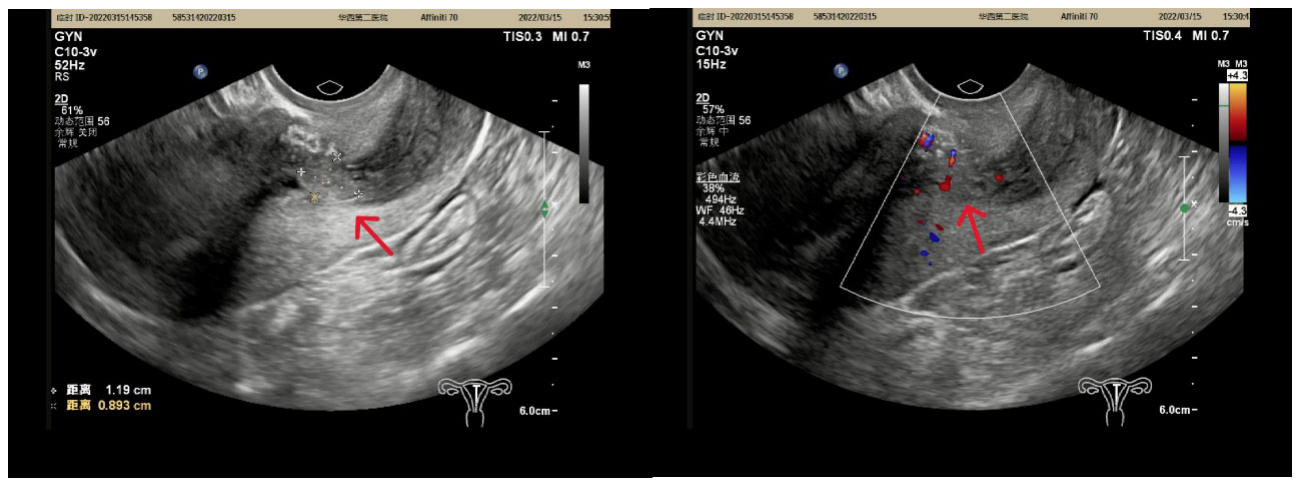
A slightly stronger echo (red arrow) of 1.2 cmx0.9 cmx1.3 cm in size was found in the lower part of the uterine cavity, with a clear border and point-like blood flow signals peripheral and inward from the echo.

A 44-year-old woman who was premenopausal presented with abnormal vaginal bleeding for one month. Physical examination indicated a polypoid mass (1.2 cm × 1.1 cm) protruding into the cervix. Then hysteroscopy and biopsy were performed. The pathological investigation was considered to be a lesion of endometrial stromal hyperplasia. The patient came to our hospital for consultation for accurate diagnosis and further treatment. Pathology consultation result revealed endometrial polyps with endometrial stromal hyperplasia, and malignment endometrial stromal tumor could not be excluded. Complete resection of the lesion was recommended. Furthermore, ultrasound showed a mass in the lower segment of the uterine body and the junction of the cervix (**Figure 1**). Therefore, the patient underwent hysterectomy and bilateral salpingo-oophorectomy, because the patient has completed the fertility.

**Figure 2:**
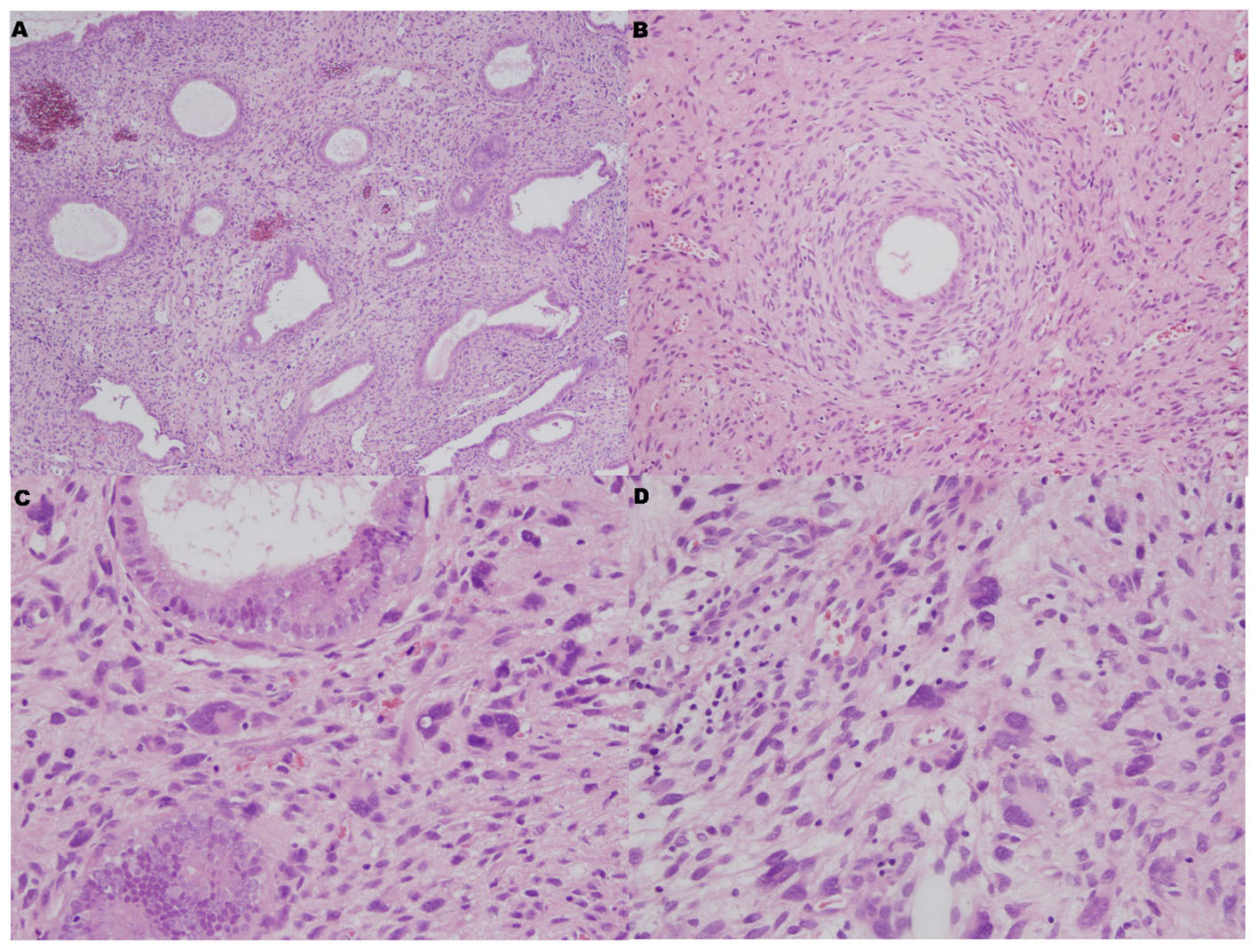
Microscopic features. **(A)** The surface and glandular epithelium was benign, but the stroma was hyperplastic (HE×40). **(B)** Focally, hypercellular stromal cuffs were observed around glands (HE×100). **(C, D)** There were numerous bizarre stromal cells that were pleomorphism, multinucleate and hyperchromatic (HE×400).

We located the lesion in the lower segment of the uterine body, and it was confined to the endometrium through gross examination. Histologically, the surface and glandular epithelium resembled proliferative endometrial epithelium, and the stroma was hyperplastic. Focally, hypercellular stromal cuffs around glands or band-like hypercellular zones beneath the surface epithelium could be seen. However, cleft-like spaces and papillary stomal fronds were absent. Tumer necrosis and myometrial invasion were not been seen. Nuclear atypia and mitotic figures could be seen in the mesenchymal cells (>2 MF/10 HPF). Extraordinarily, there were numerous bizarre stromal cells that were pleomorphism, multinucleate and hyperchromatic (**Figure 2**).

**Figure 3:**
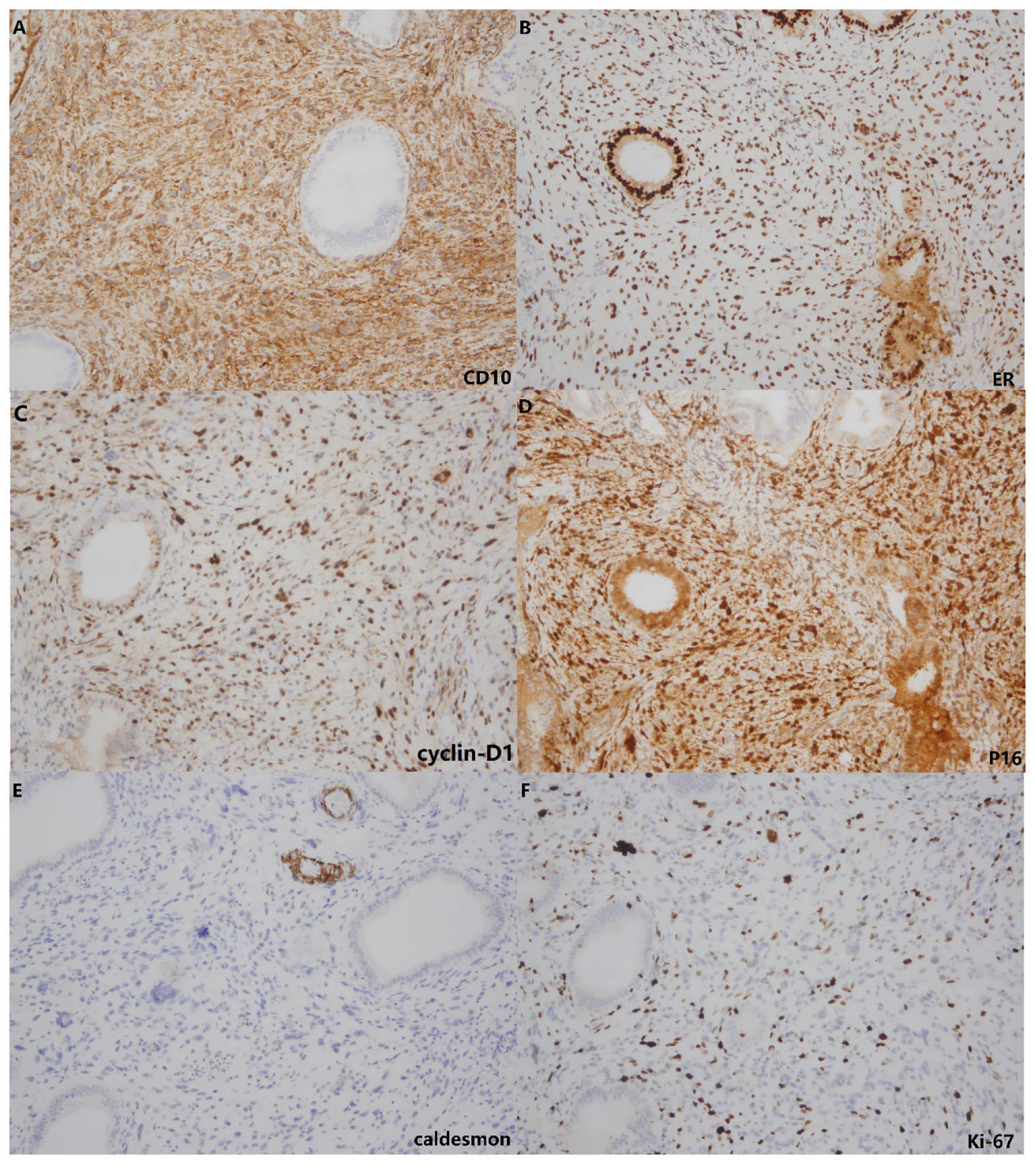
Immunohistochemistry. The stromal cells showed positive staining for CD10 **(A)**, ER **(B)**, cyclin-D1 **(C)** and P16 **(D)** but lacked staining for caldesmon **(E)**. The Ki-67 index **(F)** was approximately 10%-20%.

**Figure 4:**
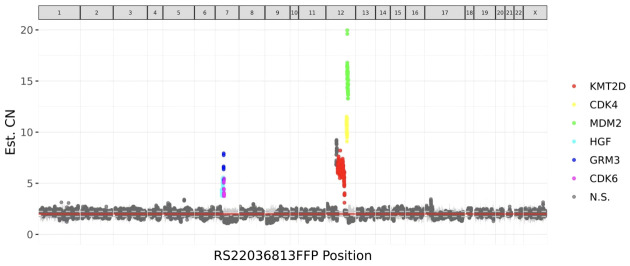
Whole-exome sequencing (WES) detected 7 mutations in the tumor, including amplification of MDM2, CDK6, CDK4, KMT2D, HGF and GRM3 and rearrangement of KMT2D. The most significant mutation was MDM2 amplification.

**Figure 5:**
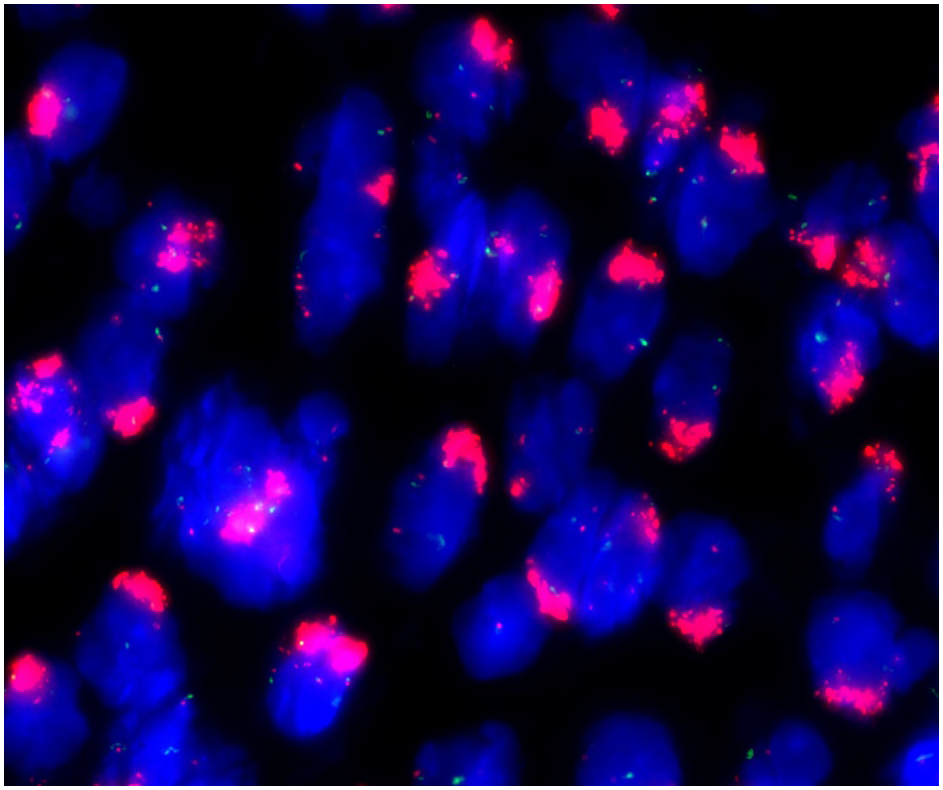
FISH. Amplification of MDM2 showed clustered signals.

These stromal tumor cells, including the bizarre cells, stained positively for vimentin, CD10, ER, PR, cyclin D1 and P16 but were immunonegative for caldesmon. The Ki-67 index was approximately 10%-20% (**Figure 3**). Whole exome sequencing (WES) was performed to analyze mutations. Seven mutations were detected in the tumor, including amplification of MDM2, CDK6, CDK4, KMT2D, HGF and GRM3 and rearrangement of KMT2D, with amplification of MDM2, CDK6 and CDK4 being most prominent (**Figure 4**). FISH (fluorescence *in situ* hybridization) was performed to verify the prominent amplification of MDM2 (**Figure 5**). At the same time, we also detected rearrangement of JAZF1, YWHAE and BCOR through FISH. These gene mutations which are frequently associated with endometrial stromal sarcoma were not found.

In conclusion, we diagnosed this polypoid lesion as adenosarcoma. The tumor was composed of a benign epithelium and malignant stroma. The interstitial substance with numerous bizarre cells was immunoreactive for CD10, ER and cyclin-D1 and showed amplification of MDM2.

By the telephone follow-up, the patient has been disease free for one year, without recurrence or metastasis.

## Discussion

As previously reported, atypical stromal cells of the female gynecologic tract have been described and reported in the vagina, vulva, and cervix; rare cases have been documented in the endometrium, especially hyperplastic polyps of the endometrium (10). Most of these findings are considered degenerative and benign, and some have postulated that these cells are of fibroblastic origin. However, the etiology and precise nature of multinucleated giant cells and atypical stromal cells in the endometrium remain unclear.

Tai and Tavassoli described a series of 15 cases of atypical stromal cells in the endometrium, 13 in endometrial polyps and 2 within the endometrial stroma, but without endometrial polyps (11). The authors suggested that these cells represent a primitive cell population originating from multipotent mesenchymal cells that have the ability to differentiate toward either smooth muscle or endometrial stromal lineages because of immunohistochemical findings of positivity of the atypical stromal cells for endometrial stromal and smooth muscle markers. In their study, all cases (100%) stained positive for vimentin, estrogen receptor, progesterone receptor, and androgen receptor, and 37% were positive for CD10. Some other case reports show a similar viewpoint. Bizarre stromal cells of an endometrial polyp that are similar to the cells of vulvovaginal polyps, as well as at nongynecologic sites, have been reported; caution to avoid mistaking these cells for malignancy has highlighted (12). Alp Usubütün et al. described a polypoid leiomyoma with bizarre nuclei and atypical endometrial stromal cells adjacent to a leiomyoma. They regarded these atypical cells as degenerative in nature and stated that they should not be mistaken for malignant cells on histologic examination, especially when examining small endometrial samples (13).

Our case, however, is different from these reports. First, rather than being distributed under the surface epithelium only, these atypical bizarre stromal cells extended wildly in the tumor, and the atypical hypercellular stromal cuffs around glands appeared focally. The Ki-67 proliferation index was approximately 10%-20%, and mitotic figures could be seen (>2 MF/10 HPF).

What’s more, whole-exome sequencing (WES) revealed amplification of MDM2, CDK4 and CDK6. Mouse double minute 2 (MDM2) is a critical negative regulator of the tumor suppressor p53, playing a key role in controlling its transcriptional activity, protein stability, and nuclear localization. Analysis of broader panels of sarcoma samples confirmed the presence of MDM2 amplification in many histological subtypes of sarcoma, including osteosarcoma, liposarcoma, lipoma, leiomyosarcoma, rhabdomyosarcoma, malignant schwannoma, fibrosarcoma, hemangiopericytoma, and malignant fibrous histiocytoma (14). The amplification of MDM2 was also validated by FISH in our case. CDK4/6 complex is a key mediator of cell cycle progression through the G1 phase, the time when a cell prepares to initiate DNA synthesis. This gene plays a key role in mammalian development and cancer (15, 16). Furthermore, previous molecular studies of adenosarcoma have revealed mutations in genes such as MDM2/CDK4, TP53, ATRX, FGFR2, KMT2C and DICER1 (6–9). Therefore, we believe that these bizarre stromal cells formed a real tumor. This atypical adenosarcoma with extensive bizarre stromal cells is first reported here.

Additionally, differential diagnosis of adenosarcoma, including benign entities such as endometrial and endocervical polyps and adenofibroma, as well as various malignant tumors, including endometrial stromal sarcoma, leiomyosarcoma, and botryoid rhabdomyosarcoma in young patients, should be considered. Based on the morphological atypia and molecular changes observed, our case did not involve a benign lesion. Immunohistochemistry suggested the tumor to be of endometrial stromal origin. However, rearrangement of JAZF1, YWHAE and BCOR, which are frequently associated with endometrial stromal sarcoma, was not found by FISH. Hence, endometrial stromal sarcoma was not diagnosed.

In summary, endometrial polyps that occur in women of childbearing age need to be considered when the mesenchymal component is significantly hyperplasic. Such a finding should not simply be considered benign when encountered bizarre nuclear stromal cells. Müllerian adenosarcoma and endometrial stromal sarcoma should be considered and differentiated through IHC, FISH and even gene sequencing. Extended clinical observation is necessary because it is unclear whether the prognosis is similar to that of a typical adenosarcoma.

## Data availability statement

The authors confirm that the data supporting the findings of this study are available within the article.

## Ethics statement

The requirement of ethical approval was waived by Ethics Committee of West China the Second Hospital of Sichuan University for the studies involving humans because this research study was conducted retrospectively according to medical record data obtained during hospitalization. The studies were conducted in accordance with the local legislation and institutional requirements. Written informed consent for participation was not required from the participants or the participants’ legal guardians/next of kin because This research study was conducted retrospectively according to medical record data obtained during hospitalization. Written informed consent was not obtained from the individual(s) for the publication of any potentially identifiable images or data included in this article.

## Author contributions

XX: Writing – original draft. YH: Writing – review & editing.
